# Cardioprotective Effects of Aconite in Isoproterenol-Induced Myocardial Infarction in Rats

**DOI:** 10.1155/2022/1090893

**Published:** 2022-12-26

**Authors:** Ziwei Xing, Chao Yang, Junyao He, Yaqian Feng, Xu Li, Cheng Peng, Dan Li

**Affiliations:** ^1^State Key Laboratory of Southwestern Chinese Medicine Resources, School of Pharmacy, Chengdu University of Traditional Chinese Medicine, Chengdu, China; ^2^National Engineering Research Center for Marine Aquaculture, Institute of Innovation & Application, Zhejiang Ocean University, Zhoushan, China

## Abstract

**Background:**

Myocardial infarction (MI) is a severe clinical condition caused by decreased or complete cessation of blood flow to a portion of the myocardium. Aconite, the lateral roots of *Aconitum carmichaelii* Debx., is a well-known Chinese medicine for treatment of heart failure and related cardiac diseases. The present study is aimed at investigating the cardioprotective effect of aconite on isoproterenol- (ISO)- induced MI.

**Methods:**

The qualitative analysis of aqueous extracts from brained aconite (AEBA) was conducted by HPLC. A rat model of MI induced by ISO was established to examine the effects of AEBA. The cardiac function was assessed by echocardiography. The serum levels of SOD, CK-MB, cTnT, and cTnI were detected to estimate myocardial injury. The pathological changes of heart tissue were evaluated by 2,3,5-triphenyltetrazolium chloride (TTC) staining, hematoxylin-eosin (HE) staining, and Masson's trichrome staining. The expressions of abnormal vascular remodeling and hypoxia-related components and the levels of inflammation-associated genes and proteins were detected by RT-qPCR, western blotting, and immunofluorescence.

**Results:**

The contents of benzoylaconine, benzoylmesaconine, benzoylhypacoitine, and hypaconitine in AEBA were 1.35 *μ*g/g, 37.35 *μ*g/g, 57.10 *μ*g/g, and 2.46 *μ*g/g, respectively. AEBA obviously improved heart function through promoting echocardiographic parameters, radial strain, and circumferential strain. The data of TTC staining, HE staining, and Masson's trichrome staining disclosed that AEBA could significantly reduce infarct size, inhibit inflammatory cell infiltration, and decrease the myocardial fibrosis. Moreover, AEBA distinctly suppressed the serum levels of SOD, MDA, CK-MB, cTnT, and cTnI in ISO-induced rats. The results of RT-qPCR indicated that AEBA inhibited the expressions of hypoxia- and inflammation-related genes, including VEGF, PKM2, GLUT-1, LDHA, TNF-*α*, IL-1*β*, IL-6, and COX2. In addition, the western blotting and immunofluorescence analyses further confirmed the results of RT-qPCR.

**Conclusion:**

In summary, our results indicate that the AEBA could improve ISO-induced myocardial infarction by promoting cardiac function, alleviating myocardial hypoxia, and inhibiting inflammatory response and fibrosis in heart tissue.

## 1. Introduction

Myocardial infarction (MI), one of the most frequent cardiovascular diseases, is the global leading cause of death [1], and there are about 197 million prevalent cases of ischemic heart disease [2]. The characteristic features of MI are insufficient blood supply and decreased oxygen provision to the cardiac tissue, eventually leading to heart failure [3]. Even though interventional cardiology techniques and drugs have been developed to reduce the risk of MI, the morbidity and mortality continue to increase in the absence of effective remedies for MI. Therefore, it is urgently needed to find new therapies to prevent MI.

The interplay between oxidative stress, inflammation, and hypoxia leads to cardiac remodeling in MI [4]. It is well recognized that oxidative stress, caused by the imbalance between the scavenging and production of oxygen radicals, aggravates maladaptive left ventricular remodeling [5]. Superoxide dismutase (SOD), an important antioxidant enzyme, is decreased in MI [6]. Additionally, oxidative stress is associated with inflammatory responses in the infarcted heart, manifested by the infiltration of inflammatory cells (such as macrophages), secretion of proinflammatory mediators [7], and activation of the inflammatory cascades [8], resulting in exacerbating abnormal vascular remodeling and cardiac dysfunction [9, 10]. Furthermore, oxidative stress and inflammation further promote myocardial hypoxia reactions. Hypoxia, as the primary inducer of cardiomyocyte injury, leads to the loss of contractile tissue and the decrease of survival cardiomyocytes [11], the increase of the ventricular wall [12], and the expansion of fibrosis [13]. Hypoxia-inducible factor-1*α* (HIF-1*α*), a critical hypoxia factor that regulates oxygen balance and promotes adaptation of cell and tissue to low oxygen concentrations, is increased during MI [14] and is regarded as one of the most important markers of myocardial hypoxia. Hence, interfering with oxidative stress, inflammation, and hypoxia might contribute to the treatment of MI.

Aconite, also called “Fuzi” in Chinese, is a processed product of Aconitum *carmichaelii* Debx. (Ranunculaceae) seed root, which is a famous traditional Chinese medicine and has been reported to possess protective effects on the cardiovascular system [15]. Water-soluble alkaloids from aconite significantly improve atrioventricular conduction and restore sinus rhythm in an animal model of heart failure [16]. Furthermore, aconite exerts protective effects on the ischemic heart by inhibiting the inflammatory response through the TLR4/NF-*κ*B pathway [17]. And aconite decoction also exhibits cardioprotective effects by promoting mitochondrial biogenesis through Sirt1/PGC-1*α* pathway [18]. However, the effects and mechanisms of aconite in MI are still unclear. Therefore, we hypothesized that aconite could exert cardioprotective effects through suppressing oxidative stress, inhibiting inflammation, and preventing adverse vascular remodeling of MI. In the present studies, we established the isoproterenol- (ISO-) induced MI model to explore the effects and underlying mechanisms of aconite on heart injury, myocardial hypoxia, and inflammation in rats.

## 2. Materials and Methods

### 2.1. Extraction of Aconite

Aconite processed by brine was acquired from Sichuan Jiangyou Zhongba Aconite Technology Development Co., Ltd. (Sichuan, China). The aqueous extract from brined aconite (AEBA) was prepared as previously reported [19]. Brined aconite was weighted and immersed in a 10 times mass of double-distilled water (1 : 10, *w*/*v*) and then was decocted for 5 h. After filtration, 8-fold amount of double-distilled water was added and decocted for another 3 h and then filtered. Subsequently, these two filtrates were mixed and concentrated to a dose of 1 g AEBA/1 g crude brined aconite, which would be used for high-performance liquid chromatography (HPLC) analysis and administration.

### 2.2. HPLC Analysis of AEBA

**Figure 1:**
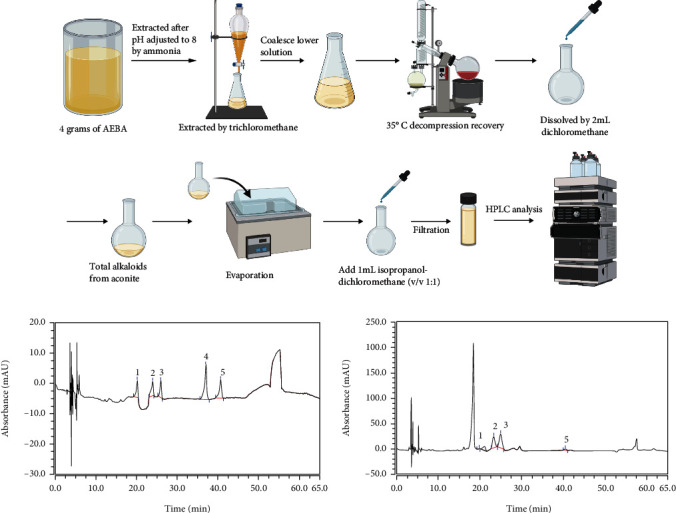
Quantification of alkaloids in AEBA by HPLC. (a) The process of sample preparation for HPLC. (b) The chromatogram of standard compounds. 1-benzoylaconine, 2-benzoylmesaconine, 3-benzoylhypacoitine, 4-mesaconine, and 5-hypaconitine. (c) The chromatogram of AEBA.

As previously reported, the AEBA was quantitatively analyzed by HPLC (U300, ThermoFisher Scientific, USA) [20]. The preparation of AEBA for HPLC was shown in Figure 1(a). The prepared 25 *μ*L AEBA was injected into an Agilent 5 TC﹣C18 (2) column (250 × 4.6 mm, 5 *μ*m) at 35°C, 235 nm with the total analysis time of 65 min. Gradient elution was carried out with acetonitrile/tetrahydrofuran (25 : 15, *v*/*v*) (A) and 0.1 mol/L aqueous ammonium acetate (B) at a flow rate of 1 mL/min as follows: in 0-48 min 85-74% B, in 48-49 min 74-65% B, in 49-58 min 65% B, and in 58-65 min 65-85% B. The benzoylaconine, benzoylmesaconine, benzoylhypacoitine, mesaconine, and hypaconitine were quantitated.

### 2.3. Experimental Animals and Protocols

24 male Wistar rats (weighing 220-240 g), obtained from SPF (Beijing) Biotechnology Co., Ltd. (Beijing, China, No. SCXK 2019-0010), were kept at SPF animal laboratory (25 ± 1°C temperatures and 55 ± 5% relative humidity) and were provided for 12-hour light/dark cycle allowed with food and water ad libitum. All animals' treatments were authorized by the Animal Ethics Committee of Chengdu University of Traditional Chinese Medicine (No. 2021-48). After one week of adaptive feeding, the rats were divided into three groups at random (*n* = 8): the control group (Control group), the ISO group (ISO group), and the ISO + AEBA group. The Control group and ISO group were intragastrically (i.g) administrated with sterile double-distilled water (10 mL/kg), and the rats in the ISO + AEBA group were treated (i.g) with the same volume of AEBA (5 g/kg/day) for continuous 7 days. On the final two days, the rats in the ISO group and ISO + AEBA group were intraperitoneally injected (i.p) with ISO (85 mg/kg/day), as well as the rats in the control group were injected with saline.

### 2.4. Echocardiography Detection

Rats were anesthetized with 3% isoflurane (RWD Life Science Co., Ltd., China) inhalation. The M-mode image from the parasternal short axis of the LV was carried out using a Vevo 3100 system (FujiFilm VisualSonics, Canada) with an MX-250 probe. Left ventricle (LV) ejection fraction (EF), LV end-diastolic dimensions (LVIDd), LV end-systolic dimensions (LVIDs), LV diastolic anterior wall thickness (LVAWd), LV systolic anterior wall thickness (LVAWs), LV diastolic posterior wall thickness (LVPWd), LV systolic posterior wall thickness (LVPWs), and fractional shortening (FS) were collected as assessments of cardiac function.

### 2.5. Echocardiographic-Based Strain Analysis of Myocardial Deformation

Parasternal short-axis B-mode images were acquired from the Vevo 3100 system. The Vevo Strain Software (FujiFilm VisualSonics) was employed to calculate the circumferential and radial strain of anterior base (AB), anterior middle (AM), anterior apex (AP), posterior base (PB), posterior middle (PM), and posterior apex (PA) [21].

### 2.6. Sample Collection and Determination of Heart Tissue

After the echocardiography detection, all rats were euthanized, and blood samples were collected. Serum was subsequently isolated and stored at ﹣80°C. The heart tissues were carefully removed after blood collection, and the auricle and surrounding connective tissue were cut out; after that, the hearts were washed with precooled saline and dried with filter paper. Then, the heart tissues were photographed and weighed. The heart tissue index (HTI) was calculated by HTI (g/g) = heart tissue weight (g)/body weight (g) × 100.

### 2.7. TTC Staining and Measurement of Myocardial Infarction Size

After the heart tissues were washed with precoded saline 3 times and wiped dry, the heart was immediately frozen in a refrigerator at ﹣20°C for 20 min and then cut into 3-5 mm slices. The slices were incubated at 37°C in 2% solution of TTC (Solarbio, China) (dissolved in phosphate buffer saline, pH 7.4) for 30 min. The infarction size of heart tissue was analyzed by ImageJ software (National Institutes of Health, USA).

### 2.8. Measurement of Serum Biomarkers

SOD and malondialdehyde (MDA) in serum were gauged by kits from the Nanjing Jiancheng Institute of Biological Engineering (Nanjing, China). The serum levels of creatine kinase isoenzyme (CK-MB), cardiac troponin T (cTnT), and cardiac troponin I (cTnI) were detected by ELISA kits (Elabscience Biotechnology Co. Ltd., China).

### 2.9. HE Staining and Masson's Trichrome Staining

Heart tissue was fixed in 4% paraformaldehyde and embedded in paraffin. 5 *μ*m slices were cut for staining with HE and Masson's trichrome staining. The extent of myocardial fibrosis was observed by Masson's trichrome staining. Collagen volume fraction (CVF) was analyzed by ImageJ: CVF = collagen area of myocardial interstitium/total field area. Six high power fields were randomly selected for each section for measuring, and the average value was taken. The histopathological images were obtained and analyzed by a digital camera (Nikon, Japan) linked to a microscope (Nikon, Japan). An injury grading score (grades 0-4) was carried out for evaluating the degree of myocardial injury as previously reported [22]. Six high power fields were randomly selected for each section for scoring, and the average value was taken.

### 2.10. Western Blot Analysis

The proteins from left ventricular tissues in each group were extracted with ice-cold RIPA buffer, which contained protease inhibitor cocktail and PMSF. And the concentration of protein was calculated by a BCA Protein Assay Kit (ThermoFisher Scientific). Equal amount of protein samples (20 *μ*g) were separated by electrophoresis in sodium dodecyl sulfate-polyacrylamide gel electrophoresis (SDS-PAGE) and then transferred to PVDF membranes (Bio-Rad, USA). The membranes were blocked with 5% skimmed milk for 1 h. Then incubated at 4°C with primary antibodies overnight. After three washes by TBST, membranes were incubated with the HRP-conjugated secondary antibody (Cell Signaling Technology, USA) for 2 h. The immunoblot signals were visualized by using chemiluminescence (ECL) substrates (ThermoFisher Scientific) in an imaging system (Tanon, China) and were analyzed by ImageJ software. The information of antibodies was shown in Table 1.

### 2.11. qRT-PCR Analysis

Total RNA of LV tissue was extracted with TRIzol Reagent (ThermoFisher Scientific) and was reverse-transcribed using Prime Script™ RT Reagent Kit (ThermoFisher Scientific). The PowerUP™ SYBR™ Green Master Mix kit (ThermoFisher Scientific) was used to amplify the cDNA samples with gene-specific primers [23, 24] (Table 2) and collected by ABI StepOnePlus PCR system (ThermoFisher Scientific). The relative mRNA expressions of genes were calculated by the 2^-△△CT^ method as described previously [25].

### 2.12. Immunofluorescence Analysis

The 5 *μ*m paraffin sections of heart tissue were deparaffinized and rehydrated, then were immersed with 10 mM citric acid buffer (pH 6.0), and heated in the water bath to recover antigenicity as described previously [26]. Then, the sections were blocked with 10% BSA for 1 h at room temperature and subsequently were stained at 4°C with primary anti-CD31 antibody (1 : 500), anti-GLUT 1 antibody (1 : 500), anti-CD68 antibody (1 : 500), and anti-angioprotein-2 antibody (1 : 500) overnight. After rinsing three times with PBS, the slides were incubated at room temperature with Alexa Flour 488 goat anti-mouse IgG (ThermoFisher Scientific) or Alexa Flour 594 goat anti-Rabbit IgG (ThermoFisher Scientific) for 1 h in darkness. Then, the sections were washed three times with PBS and treated with DAPI to counterstain the nucleus for 20 minutes. The stained slides were examined with a confocal fluorescence microscope (Olympus, Japan).

### 2.13. Statistical Analysis

All data were presented as mean ± S.D. and were analyzed with GraphPad Prism 8.2.1 software (San Diego, USA). The intergroup comparisons were evaluated by one-way ANOVA. And the results were considered statistically significant when *p* values are less than 0.05.

## 3. Results

### 3.1. Quantification of Alkaloids in AEBA

Figure 1(a) exhibits the process of sample pretreatment for HPLC. The chromatogram of standard compounds was shown in Figure 1(b), and the contents of benzoylaconine, benzoylmesaconine, benzoylhypacoitine, and hypaconitine in the AEBA were, respectively, 1.35 *μ*g/g, 37.35 *μ*g/g, 57.10 *μ*g/g, and 2.46 *μ*g/g (Figure 1(c)), while the mesaconine was not detected.

### 3.2. AEBA Improves Echocardiography Parameters of ISO-Induced Rats

**Figure 2:**
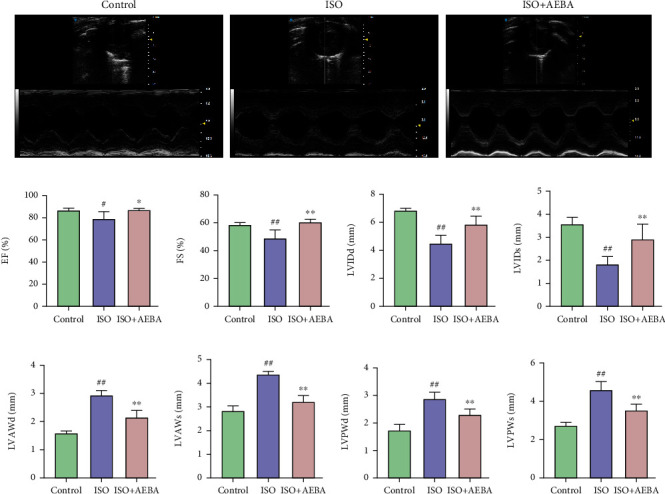
AEBA improved heart function in ISO-induced rats. (a) The representative short-axis M-mode images of echocardiography. (b) The change of EF. (c) The change of FS. (d) The change of LVIDd. (e) The change of LVIDs. (f) The change of LVAWd. (g) The change of LVAWs. (h) The change of LVPWd and (i) LVPWs. *n* = 8. ^#^*p* < 0.05 and ^##^*p* < 0.01 vs. control group; ^∗^*p* < 0.05 and ^∗∗^*p* < 0.01 vs. model group.

To investigate the therapeutic effects of AEBA on ISO-induced MI, echocardiography was used for the evaluation of cardiac function. As demonstrated in Figure 2, AEBA significantly increased EF (*p* < 0.05) (Figure 2(b)), FS (*p* < 0.01) (Figure 2(c)), LVIDd (*p* < 0.01) (Figure 2(d)), and LVIDs (*p* < 0.01) (Figure 2(e)) in ISO-induced MI rats. Similarly, compared with the ISO group, the LVAWd (Figure 2(f)), LVAWs (Figure 2(g)), LVPWd (Figure 2(h)), and LVPWs (Figure 2(i)) were significantly relieved (*p* < 0.01) after AEBA treatment, suggesting that AEBA could ameliorate cardiac function in ISO-induced MI.

### 3.3. AEBA Ameliorates ISO-Induced Myocardial Deformation in Rats

**Figure 3:**
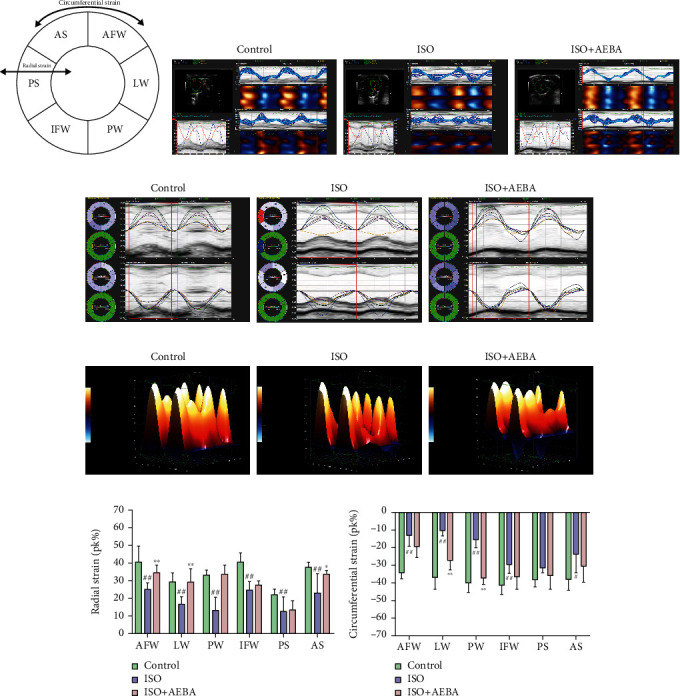
AEBA improved strain analysis of myocardial deformation. (a) Regions of LV for strain assessment. (b) The interface of strain analysis. (c) The representative images of radial and circumferential strain. (d) 3D reconstruction of radial strain. (e) Quantification of radial and (f) circumferential strain expressed as Pk (%). *n* = 8. ^#^*p* < 0.05 and ^##^*p* < 0.01 vs. control group; ^∗^*p* < 0.05 and ^∗∗^*p* < 0.01 vs. model group.

We further analyzed the myocardial function of rats by using strain analysis software. As shown in Figure 3(a), the short axis of the LV was segmented into six parts by the strain software automatically. Figures 3(b)–3(d) illustrate the changes in myocardial strain in MI rats from both planar and 3D, which were statistically represented by Figures 3(e) and 3(f). Compared with the Control group, the data showed that the radial strain in the ISO group was decreased, and the circumferential strain was increased, indicating that the ISO caused insufficient cardiac function in rats. While the AEBA significantly increased the radial strain of AFW (*p* < 0.01), LW (*p* < 0.01), and AS (*p* < 0.05), the circumferential strain of LW and PW (Figures 3(e) and 3(f)) was significantly decreased (*p* < 0.01), indicating the enhancement of heart function. These data suggested that the changes of radial and circumferential deformation of the LV were improved by the treatment of AEBA.

### 3.4. AEBA Improves Heart Parameters and Relieves Heart Injury

**Figure 4:**
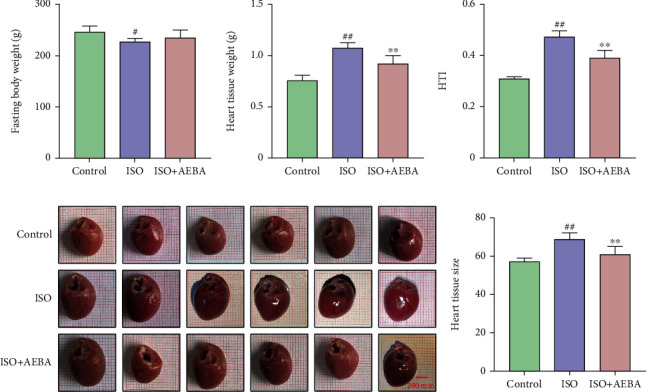
Aconite alleviated infarct area size and heart-to-body ratio. (a) Fasting body weight. (b) Heart tissue weight. (c) Heart tissue index. (d) Cardiac morphology. (e) Relative size of the heart. Scale bar = 200 *μ*m. *n* = 8. ^#^*p* < 0.05 and ^##^*p* < 0.01 vs. control group; ^∗^*p* < 0.05 and ^∗∗^*p* < 0.01 vs. model group.

Next, the cardiac parameters of rats were measured. AEBA alleviated ISO-induced body weight loss (Figure 4(a)), effectively inhibited (*p* < 0.01) cardiac hypertrophy (Figure 4(b)), and decreased (*p* < 0.01) heart tissue index (Figure 4(c)). The hearts of AEBA-treated rats were significantly smaller (*p* < 0.01) than the ISO group by measuring heart morphometric (Figures 4(d) and 4(e)).

**Figure 5:**
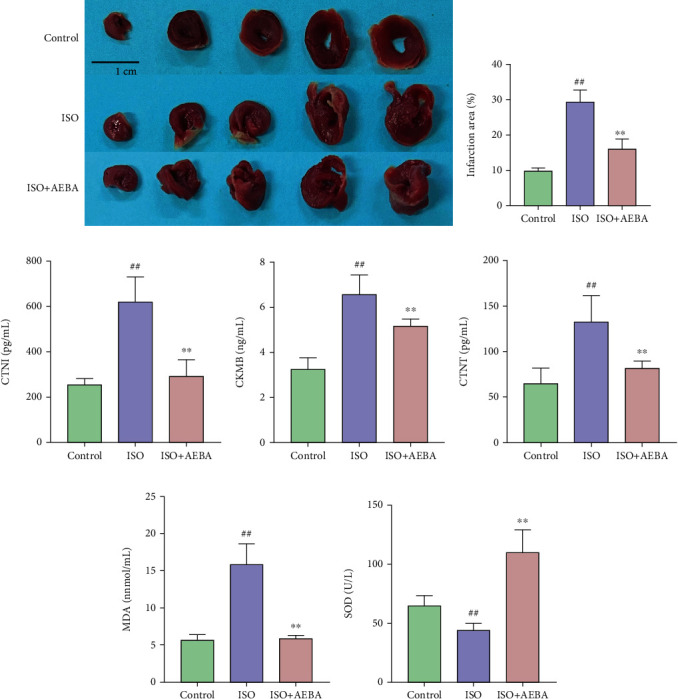
AEBA decreased the infarction area of heart tissue and serum enzyme index in ISO-induced MI of rats. (a) Representative images of TTC staining. Scale bar = 1 cm. (b) Infarction area of heart tissue in ISO-induced rats. The levels of CTNI (c), CK-MB (d), CTNT (e), MDA (f), and SOD activity (g) in serum of ISO-induced rats. *n* = 8. ^#^*p* < 0.05 and ^##^*p* < 0.01 vs. control group; ^∗^*p* < 0.05 and ^∗∗^*p* < 0.01 vs. model group.

In addition, planimetric determination of infarct sizes with TTC showed different degrees of infarction in the ISO group, while AEBA could significantly reduce the infarction area in ISO-induced MI rats (*p* < 0.01) (Figures 5(a) and 5(b)). Furthermore, the serum levels of CK-MB, cTnI, cTnT, and MDA and the activity of SOD were measured to verify the therapeutic effects of AEBA on MI. The results showed that AEBA could significantly reduce (*p* < 0.01) the levels of cTnI (Figure 5(c)), CK-MB (Figure 5(d)), and cTnT (Figure 5(e)), MDA (Figure 5(f)) and increase the activity of SOD (Figure 5(g)) in serum of ISO-treated rats. These results showed that AEBA could significantly reverse the ISO-induced oxidative stress damage to heart dysfunction.

### 3.5. AEBA Alleviated ISO-Induced Pathological Damage in Rats

**Figure 6:**
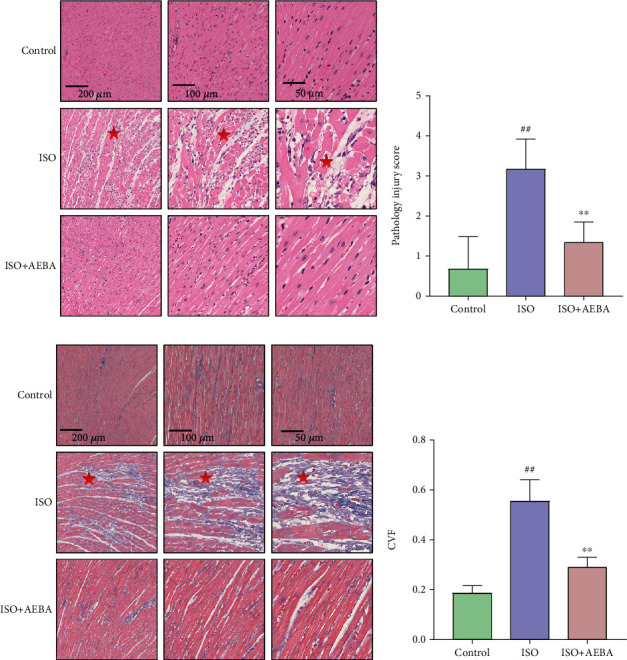
AEBA alleviated ISO-induced pathological damage in rats. (a) HE staining of heart tissue of rats in each group. (from left to right, scale = 200 *μ*m, 100 *μ*m, 50 *μ*m). (b) Pathology injury score of HE staining, *n* = 6. (c) Masson's trichrome staining of heart tissue. (from left to right, scale bar = 200 *μ*m, 100 *μ*m, 50 *μ*m). (d) The CVF of each group. *n* = 6. ^#^*p* < 0.05 and ^##^*p* < 0.01 vs. control group; ^∗^*p* < 0.05 and ^∗∗^*p* < 0.01 vs. model group.

To visualize the extent of damage to cardiac tissue following MI, HE and Masson's trichrome staining were used. HE staining showed that the cardiomyocytes were neatly arranged with clear spaces, and oval nucleus were located in the center of the cell without damage in the Control group, while the heart tissue of the ISO group exhibited myocardial necrosis and edema, inflammatory cells' infiltration, and membrane damage (*p* < 0.01) (Figures 6(a) and 6(b)). In contrast, myocardial tissue arrangement was denser, and AEBA significantly reduced (*p* < 0.01) the infiltration of inflammatory cells (Figures 6(a) and 6(b)). Furthermore, as shown in Figures 6(c) and 6(d), the degree of myocardial fibrosis and the positive areas of myocardial fiber staining were significantly increased (*p* < 0.01) in the ISO group, while collagen deposition and the CVF were significantly reduced (*p* < 0.01) after AEBA treatment. These findings revealed that AEBA could alleviate the inflammatory infiltration and myocardial fibrosis in heart tissue of ISO-induced rats.

### 3.6. AEBA Ameliorates Abnormal Vascular Remodeling and Hypoxia in ISO-Induced Rats

**Figure 7:**
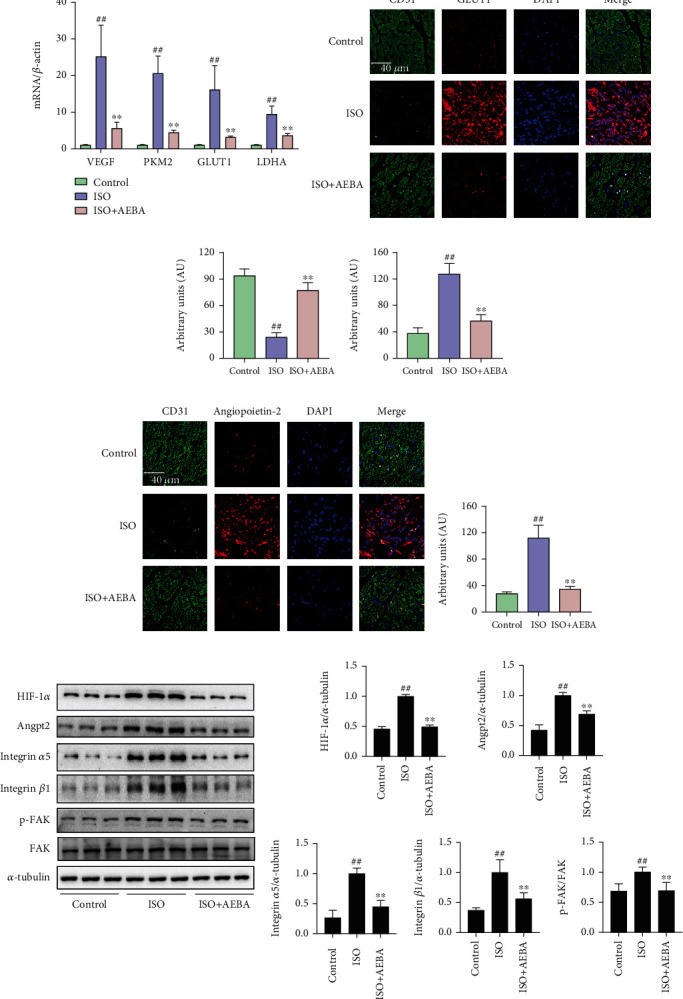
AEBA ameliorates ISO-induced abnormal vascular remodeling and hypoxia in rats. (a) The mRNA expression of VEGF, PKM2, GLUT-1, and LDHA in the LV of heart tissue. (b) Representative immunofluorescent staining images of GLUT-1 (red), CD31 (green), and nuclei (blue) in the heart tissue sections. Scale bar = 40 *μ*m. Mean fluorescence intensity of (c) CD31 and (d) GLUT-1 analyzed by ImageJ. (e) Representative immunofluorescent staining images of Angpt2 (red), CD31 (green), and nuclei (blue) in the heart tissue sections. Scale bar = 40 *μ*m. (f) Mean fluorescence intensity of Angpt2. (g) The levels of HIF1-*α*, Angpt2, integrin *α*5, integrin *β*1, FAK, and p-FAK were detected by western blotting and analyzed by ImageJ. *n* = 6. *p* < 0.05 and ^##^*p* < 0.01 vs. control group; ^∗^*p* < 0.05 and ^∗∗^*p* < 0.01 vs. model group.

The reduction of blood supply predisposes cardiomyocytes to death or dysfunction [27], which aggravates abnormal vascular remodeling and cardiac hypoxia following MI. RT-qPCR results indicated that the mRNA expressions of VEGF, PKM2, GLUT-1, and LDHA in ISO-induced rats were remarkably increased (*p* < 0.01) (Figure 7(a)) and were decreased (*p* < 0.01) after AEBA treatment. The immunofluorescent staining displayed that the expression of vascular marker CD31 was significantly decreased (*p* < 0.01), and the levels of hypoxia markers including GLUT1 and Angprotein-2 (Angpt-2) were elevated (*p* < 0.01) in myocardial tissue of ISO-induced rats, while AEBA treatment significantly increased the expression of CD31 (*p* < 0.01) and inhibited the levels of GLUT-1 and Angpt-2 (*p* < 0.01) (Figures 7(b)–7(f)). The expression of HIF1-*α* was increased in heart tissue of MI rats, and AEBA significantly reversed it (*p* < 0.01) (Figure 7(g)). Moreover, the levels of Angpt-2, integrin *α*5, integrin *β*1, and p-FAK were significantly increased (*p* < 0.01) in heart tissue of MI rats, which were significantly reduced (*p* < 0.01) by AEBA treatment (Figure 7(g)). Therefore, AEBA could alleviate ISO-induced MI by improving abnormal vascular remodeling and inhibiting hypoxia response in heart tissue.

### 3.7. AEBA Alleviated Inflammatory Response in ISO-Induced Rats by Inhibiting MAPK Signals

**Figure 8:**
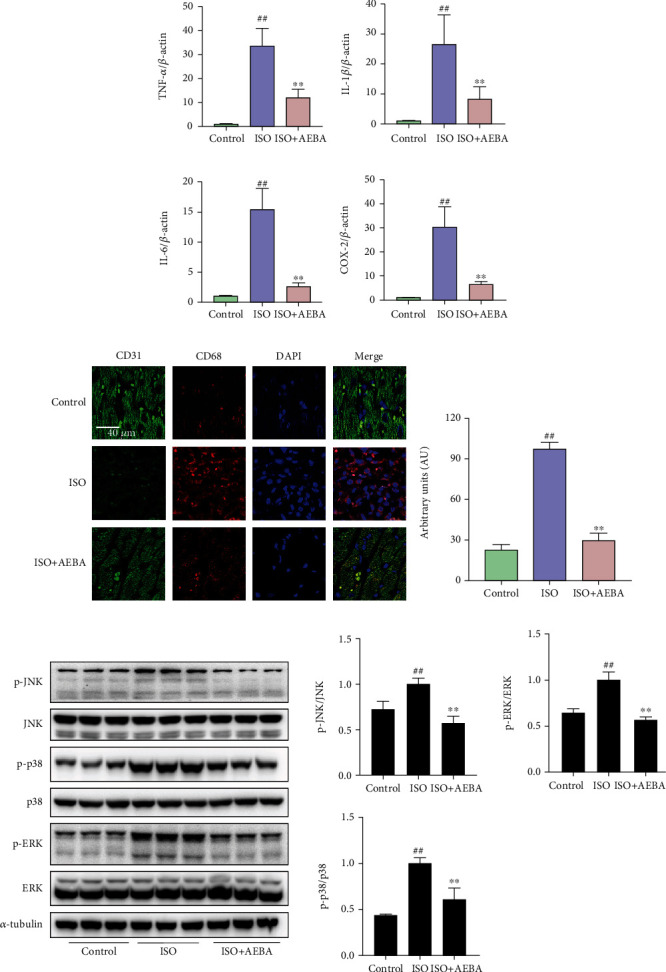
AEBA prevented cardiac inflammation in ISO-induced rats. The mRNA expressions of (a) TNF-*α*, (b) IL-1*β*, (c) IL-6, and (d) COX2 in the heart tissue. (e) Representative immunofluorescent staining images of CD68 (red), CD31 (green), and nuclei (blue) in the heart tissue sections. Scale bar = 40 *μ*m. (f) Mean fluorescence intensity of CD68. (g) The levels of MAPKs signals were analyzed by ImageJ. *n* = 6. *p* < 0.05 and ^##^*p* < 0.01 vs. control group; ^∗^*p* < 0.05 and ^∗∗^*p* < 0.01 vs. model group.

Inflammation, another critical factor, contributes to the pathological process of MI [28]. The mRNA expressions of inflammatory factors, including IL-1*β*, COX2, IL-6, and TNF-*α*, were significantly increased (*p* < 0.01) in myocardial tissue of ISO-induced MI rats, while AEBA remarkably inhibited (*p* < 0.01) these mRNA expressions (Figures 8(a) and 8(d)). The expression of macrophage marker CD68 was increased (*p* < 0.01) in myocardial tissue (Figures 8(e) and 8(f)) and was decreased (*p* < 0.01) by the treatment of AEBA. Furthermore, the expressions of p-JNK, p-P38, and p-ERK1/2 were observably increased (*p* < 0.01) in myocardial tissue after ISO injection, and AEBA notably suppressed (*p* < 0.01) the phosphorylation levels of JNK, P38, and ERK (Figure 8(g)). The above data indicated that AEBA could suppress inflammatory cells' infiltration into myocardial tissue and inhibit the activation of MAPK signaling pathway, resulting in mitigating inflammatory response in ISO-treated heart tissue.

## 4. Discussion

MI is a life-threatening disease, and its clinical symptoms are manifested by loss of myocardial contractility, increase of myocardial load, and changes in left ventricular size, geometry, and thickness [29]. Consistent with previous reports, ISO treatment resulted in pathological myocardial hypertrophy, which is characterized by distended cardiomyocytes and thickened ventricular walls in MI rats [30]. After AEBA treatment, the EF and FS were increased, and the myocardial wall thickness and geometric shape of hearts were significantly decreased in ISO-induced rats. Strain echocardiography, a strong predictor of cardiac disease, provides additional cardiac information than echocardiography alone, determines myocardial function through marking the endocardial and epicardial borders, tracking the shortening of myofiber length during systole and the degree of systolic radial myofiber thickening [31], and can pinpoint left ventricular dysfunction prior to the decrease of LVEF [32]. Strain analysis revealed that AEBA improved left ventricular deformation, which was reduced by ISO treatment. These results suggest that AEBA pretreatment could alleviate impairment of left ventricular systolic function and decrease myocardial thickness.

cTNT, cTNI, and CK-MB are highly sensitive for the early detection of cardiomyocyte injury in chest pain patients [33]. Previous study found that ISO leads to the production of oxidative free radicals which causes irreversible disruption of the cardiac myocyte membrane and myocardial cell death, resulting in the leakage of cardiac injury markers [34]. Other features of MI are degeneration, fatty vacuolation, inflammatory infiltration, myolysis, and atrophy of myocardial fibers [28]. Our results showed that AEBA could reduce the heart infarction area and suppress the serum levels of cardiac injury markers, including cTnI, CK-MB, and cTnT in ISO-induced MI rats, suggesting that AEBA maintains the structural and functional integrity of the cardiomyocyte membrane as well as its permeability. Oxidative stress induced by ISO is a critical mediator in the pathological progression of MI that triggers myocardial fibrosis and necrosis of cardiac muscle [35]. The production of oxidative free radicals affects unsaturated fatty acids on cell membranes, causing peroxidation of membrane lipids, which in turn leads to damage to cardiomyocytes and the formation of lipid peroxides [36]. MDA, a major metabolite of oxidative free radicals, reflects the degree of peroxidation of myocardial tissue. And SOD as a vital antioxidant enzyme can improve myocardial oxidative stress injury by scavenging oxygen free radicals [6, 37]. AEBA significantly decreased the content of MDA and increased the serum SOD activity, which might contribute to its effects on alleviating the injury degree of inflammation and fibrosis of the heart tissue in ISO-induced MI rats. These shreds of evidence indicate that AEBA could protect cardiomyocytes and alleviate cardiac pathological remodeling by attenuating oxidative stress.

Hypoxia is the primary inducer of cardiomyocyte injury and further contributes to triggering abnormal vascular remodeling and cardiac dysfunction [38]. The current study suggests that ISO leads to increased oxygen demand and reduced oxygen supply, resulting in severe myocardial hypoxia [39]. Clinical research found that hypoxia activates the myocardial regulating the adaptive metabolic program to maintain the energy demands between cardiac glycolytic metabolism and redox homeostasis by accumulating HIF-1*α* in patients with cyanotic congenital heart disease [40]. Under hypoxic conditions, there is a considerable escalation in HIF-1*α* level in the heart [41], and the glycolysis regulatory molecules such as GLUT1, LDHA, and PKM2 are elevated [42]. In our study, AEBA significantly inhibited the ISO-induced increased expression of HIF-1*α* and the levels of related genes. In addition, hypoxia-induced HIF-1 increase in patients with ischemic heart disease promotes the gene transcription of angiogenic factors (including VEGF and Angpt2), which further causes vascular remodeling [43]. VEGF as a direct target of HIF1-*α* can stimulate the Angpt2 expression that promoted abnormal vascular remodeling through activating integrin *α*5*β*1 signaling and the phosphorylation of FAK [44]. After AEBA treatment, the levels of VEGF and Angpt2 and its downstream molecules were suppressed in ISO-induced heart tissue. These results indicate that AEBA could alleviate hypoxia in MI via inhibiting HIF-1*α*.

Irreversible cardiac damage caused by ischemia and hypoxia facilitates the activation of macrophages and further leads to a series of inflammatory responses [9, 45]. Targeting anti-inflammatory strategies aimed at reducing infarct size and attenuating injury following MI have been applied to the clinical therapy [9]. ISO administration stimulates the infiltration of macrophages and triggers the transcription of proinflammatory genes, which are known to play pivotal roles in the inflammation in MI [46]. Notably, the number of macrophages' marker CD68 and the mRNA levels of proinflammatory cytokines including IL-1*β*, IL-6, TNF-*α*, and COX-2 were inhibited by AEBA. Furthermore, the MAPK signals activated by ISO exacerbate the inflammatory cascades ending in the heart tissue [47, 48]. The MAPK signaling is involved in the immune response that promotes the pathological process of cardiac hypertrophy, ischemia/reperfusion injury, and pathological remodeling [49, 50]. AEBA treatment markedly suppressed the phosphorylation of JNK, ERK, and p38, subsequently reducing the expression of proinflammatory cytokines. Thus, AEBA could mitigate the inflammatory damage to cardiac tissues by inhibiting the MAPK signaling pathway.

There are several limitations concerning this study that should be noticed. Firstly, an explicit demonstration that how AEBA reduces inflammatory damage in cardiac tissue by inhibiting the MAPK signaling pathway needs to be further explored. Secondly, further studies are needed to clarify the potential mechanism of AEBA in preventing adverse vascular remodeling. Finally, there is a need to explore the components responsible for the cardioprotective effects of AEBA.

## 5. Conclusions

In summary, our results indicate that the AEBA exerts cardioprotective effects through promoting systolic and diastolic function of the cardiac, reducing the leakage of cardiac injury enzymes, inhibiting the infiltration of inflammatory cells, and suppressing the collagen deposition in ISO-induced MI rats, and these beneficial effects might be related to the capacity of aconite in antioxidative stress, anti-inflammation, and antihypoxia via the HIF1-*α* and MAPK signaling pathways. These findings may help to further justify the cardioprotective effects of aconite.

## Figures and Tables

**Table 1:** Antibody information used in the western blot analysis.

Antibodies	Source	Production company	Catalog numbers
Anti-phospho-JNK (Thr183/Tyr185)	Rabbit	Cell Signaling Technology	#4668
Anti-JNK	Rabbit	Cell Signaling Technology	#9252
Anti-phospho-p38 (Thr180/Tyr182)	Rabbit	Cell Signaling Technology	#4511
Anti-p38	Rabbit	Cell Signaling Technology	#8690
Anti-phospho-ERK (Thr202/Tyr204)	Rabbit	Cell Signaling Technology	#4370
Anti-ERK	Rabbit	Cell Signaling Technology	#4695
Anti-integrin *α*5	Rabbit	ABclonal	A19069
Anti-integrin *β*1	Rabbit	ABclonal	A2217
Anti-p-FAK (Tyr576/577)	Rabbit	Cell Signaling Technology	#3281
Anti-FAK	Rabbit	Cell Signaling Technology	#3285
Anti-HIF1*α*	Rabbit	ABclonal	A11945
Anti-GLUT1	Rabbit	Affinity	#AF5462
Anti-CD68	Rabbit	ABclonal	A6554
Anti-CD31	Mouse	Proteintech	66065-2-Ig
Anti-Angpt2	Rabbit	Affinity	#DF6137
*α*-Tubulin	Mouse	ABclonal	AC012
Anti-rabbit IgG, HPR-linked antibody	Goat	Cell Signaling Technology	#7074
Anti-mouse IgG, HPR-linked antibody	Horse	Cell Signaling Technology	#7076
Alexa Flour 488 goat anti-mouse IgG	Goat	ThermoFisher Scientific	A11029
Alexa Flour 594 goat anti-rabbit IgG	Goat	ThermoFisher Scientific	A11037

**Table 2:** Sequences of primers used in the gene expression analysis.

Gene	Primer sequence (5′ to 3′)
TNF-*α*	F: TACTCCCAGGTTCTCTTCAAGG
	R: GGAGGCTGACTTTCTCCTGGTA
IL-6	F: GAGTTGTGCAATGGCAATTC
	R: ACTCCAGAAGACCAGAGCAG
Cox2	F: CGGAGGAGAAGTGGGGTTTAGGAT
	R: TGGGAGGCACTTGCGTTGATGG
IL-1*β*	F: CACCTCTCAAGCAGAGCACAG
	R: GGGTTCCATGGTGAAGTCAAC
GLUT1	F: CTGGCTGCTGGATAGAATGAG
	R: TGTTGGGAGTCAATGGTGTC
VEGF	F: TCACCAAAGCCAGCACATAG
	R: TTTCTCCGCTCTGAACAAGG
PKM2	F: TCCCATTCTCTACCGACCTG
	R: TTCAGTGTGGCTCCCTTCTT
LDHA	F: GTCAGCAAGAGGGAGAGAGC
	R: CACTGGGTTTGAGACGATGA
*β*-Actin	F: GAAGTGTGACGTTGACATCCG
	R: TGCTGATCCACATCTGCTGGA

## Data Availability

The data used to support the findings of this study are available from the corresponding authors upon request.
